# Pre-donation BMI and preserved kidney volume can predict the cohort with unfavorable renal functional compensation at 1-year after kidney donation

**DOI:** 10.1186/s12882-019-1242-0

**Published:** 2019-02-08

**Authors:** Kazunobu Shinoda, Shinya Morita, Hirotaka Akita, Satoshi Tamaki, Ryohei Takahashi, Hidaka Kono, Hiroshi Asanuma, Eiji Kikuchi, Masahiro Jinzaki, Ken Nakagawa, Mototsugu Oya

**Affiliations:** 1 Department of Urology, Keio University School of Medicine, Tokyo, 160-8582 Japan; 2 Department of Nephrology, Toho University Faculty of Medicine, 7-5-23 Omorinishi Ota-ku, Tokyo, 143-0015 Japan; 3 Department of Diagnostic Radiology, Keio University School of Medicine, Tokyo, 160-8582 Japan; 4 Department of Urology, Tokyo Dental College Ichikawa General Hospital, Chiba, 272-8513 Japan

**Keywords:** Kidney transplant donor, Renal function compensation, CT volumetry, BMI, Preserved kidney volume

## Abstract

**Background:**

The magnitude of renal function recovery after kidney donation differs in donors with a heterogeneous background. Preoperative assessment of candidates with potentially unfavorable renal functional compensation is critical when baseline kidney function is marginal. We explored the significance of preserved kidney volume (PKV) and known preoperative risk factors for the prediction of unfavorable renal function compensation.

**Methods:**

We enrolled 101 living donors for whom a 1-mm sliced enhanced computed tomography scan was performed preoperatively and clinical data could be collected up to 1 year after donation. The donors whose estimated glomerular filtration rate (eGFR) at 1 year after donation was 70% or higher of baseline eGFR were assigned to the “favorable renal compensation” group and the others to the “unfavorable renal compensation” group.

**Results:**

Age, sex, and preoperative serum uric acid level were not significant predictors for “unfavorable renal compensation.” Multivariable logistic regression analysis revealed that body mass index (BMI) and body surface area (BSA)-adjusted PKV were independent preoperative risk factors for “unfavorable renal compensation” (adjusted odds ratio, 1.342 and 0.929, respectively). Hypertension and preoperative eGFR were not independent predictors when adjusted with BMI and BSA-adjusted PKV. Receiver operative characteristic analysis revealed that the predictive equation with the two independent predictors yielded a good accuracy to detect donor candidates with unfavorable renal functional compensation (area under the curve = 0.803), and the optimal cut-off values were identified as 23.4 kg/m^2^ for BMI and 107.3 cm^3^/m^2^ for BSA-adjusted PKV.

**Conclusions:**

BMI and BSA-adjusted PKV may be useful to select candidates with potentially unfavorable renal function compensation before kidney donation.

## Background

The prognosis of donors for living-related kidney transplantation has been of great concern for physicians, because otherwise they would be potentially healthy individuals. This issue has been argued and conflicting conclusions exist [1–6]. In several years ago, the majority argument was that cardiovascular disease risk or overall mortality risk in kidney transplant donors were comparable to those in matched non-donor population [1–3]. However, a recent report using a Norwegian cohort, which clearly disclosed exclusion criteria (e.g., age, body mass index [BMI], blood pressure [BP], BP medication, diabetes, and cardiovascular disease) of the donor and control cohorts, demonstrated that the risk of all-cause and cardiovascular deaths in the kidney donors was relatively higher than that in the controls. Furthermore, it showed that the risk of end-stage renal disease (ESRD) was unexpectedly much higher than in the controls (hazard ratio, 11.38) [4]. In the population of mixed races, black men, especially young black men, have the highest risk of developing ESRD in the late phase after donation [5, 6]. Attaining a healthy condition and not developing chronic kidney disease (CKD) stage in lifetime for kidney donors is mandatory for physicians who grant permission to become a kidney donor. When baseline kidney function of donor candidates is marginal, it is very important to assess which candidates have a potential of unfavorable renal function compensation before kidney donation.

Several reports have proposed predicting factors for post-donation kidney function in kidney donors [5–8]. Age, obesity, hypertension, albuminuria, or pre-donation kidney function are known risk factors for unfavorable renal function after donation. Many reports set the primary endpoint as net estimated glomerular filtration rate (eGFR) at some points postoperatively [6–8] and one report discussed the associated factors with the recovery of renal function after kidney donation [9]. Few reports have investigated preoperative factors that possibly affect the magnitude of renal function recovery after kidney donation. In the present study, we aimed to investigate the significant preoperative factors that could predict the candidates with a potential of unfavorable renal function compensation; the investigated outcome is not kidney function but the magnitude of renal function recovery after the donation. We used a new preoperative factor, calculated kidney volume of 3-dimensional (3-D) reconstruction by thin-sliced computed tomography (CT) scan, as a candidate of significant risk factor instead of split renal function by scintigraphy because it was recently reported that CT volumetry is superior to renal scintigraphy for prediction of preserved kidney function [10].

## Methods

### Ethics statement

This study was conducted in compliance with the ethical standards of the Declaration of Helsinki and was approved by the research ethics committee of Keio University School of Medicine (authorization number, 2018–0130). The research ethics committee approved waiving informed consent from participants because this was a retrospective and noninvasive study.

### Patient selection

Between April 2000 and September 2014, we performed 144 donor nephrectomies for living-related kidney transplantation. Among them, we retrospectively enrolled 101 patients for whom 1-mm thin-sliced enhanced CT was performed before kidney donation. All patients were Japanese. Clinical data were collected periodically at least up to a year postoperatively. All surgeries were performed via a laparoscopic procedure at our institute. We excluded patients with diabetes or glucose intolerance, those with urinary albumin excretion was ≥30 mg/gCreatinine (Cr), and those with preceding coronary or other heart diseases.

### CT volumetry

**Fig. 1:**
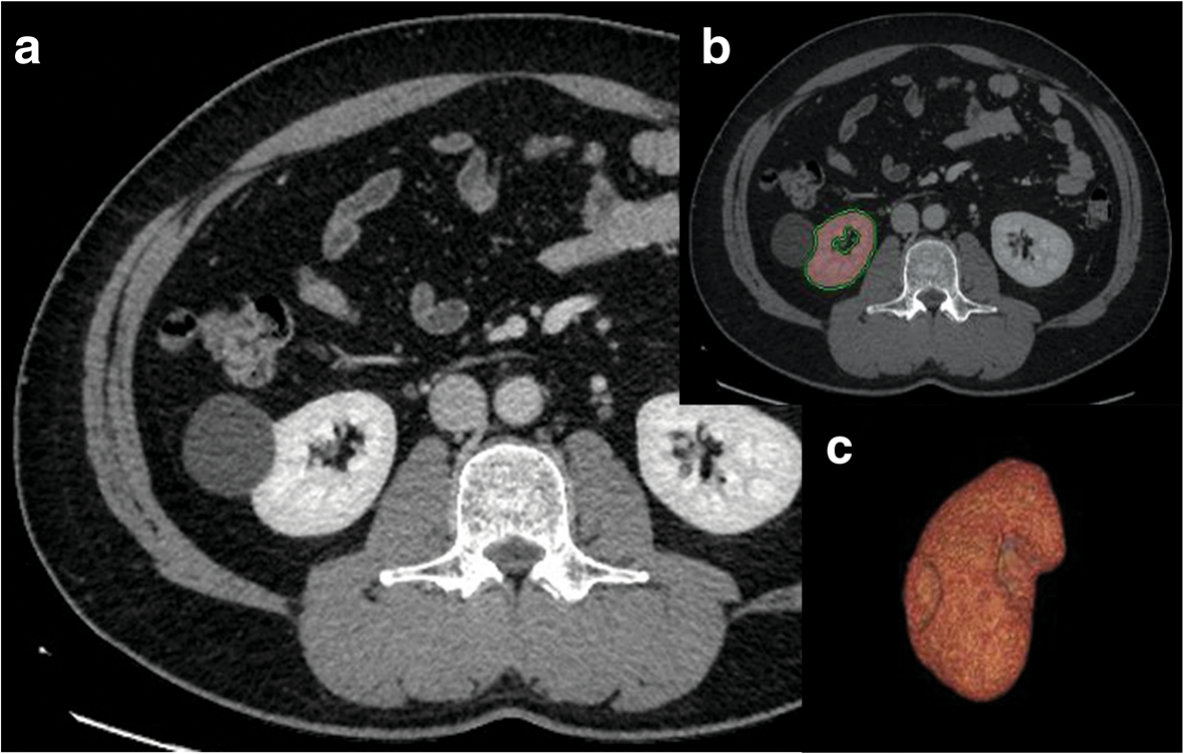
Representative semiautomatic calculation of kidney volume using 3-D reconstruction methods. **a** An axial plane of the nephrographic phase in a representative patient. **b** The kidney contour was semiautomatically drawn with pixels of the same CT values being united on an axial plane. **c** 3-D reconstructed image was created automatically and the volumetry was performed

Preoperative dynamic CT was performed using 16- or 64-multidetector CT scanners (LightSpeed Pro16, LightSpeed VCT, BrightSpeed, and Discovery CT750 HD; GE Healthcare, Waukesha, WI, USA) with a bolus-tracking technique. Iodinated contrast media (iohexol, Omnipaque 300; Daiichi Sankyo, Tokyo, Japan) at a dose of 2 mL/kg was injected for 30 s using a power injector following unenhanced CT scan. The region-of-interest cursor for bolus tracking was placed in the aorta at the celiac axis level. Acquisition of the corticomedullary phase started 12 s after reaching the threshold of 150 Hounsfield units. The nephrographic phase (Fig. 1a) was acquired at 55 s after acquisition of the corticomedullary phase. The excretory phase started 8 min after contrast injection. The nephrographic phase images were transferred and analyzed on a dedicated workstation (AW server 2.0–12.0; GE Healthcare). The kidney contour was semiautomatically drawn after a mouse click on the renal parenchyma with pixels of the same CT values being united on an axial plane (Fig. 1b). The software semiautomatically reconstructed a 3-D image of the kidney by accumulating 1-mm sliced axial images and calculated the kidney volume. Structures other than renal parenchyma (e.g., renal cyst, renal vessels, or collecting system) were eliminated from the reconstructed kidney (Fig. 1c). We measured total kidney volume (cm^3^) and preserved kidney volume (PKV). We also calculated body surface area (BSA)-adjusted PKV (BSA-adjusted PKV): BSA-adjusted PKV = PKV/BSA (cm^3^/m^2^).

### Renal function assessment and study design

Preoperative renal function was assessed by 24-h creatinine clearance (CCr) evaluation and eGFR calculation. CCr was calculated by the following equation: CCr (mL/min/1.73m^2^) = (urine Cr [mg/dL] × 24-h urine volume [mL])/24/60/serum Cr (mg/dL) × 1.73/BSA. BSA = (weight)^0.425^ × (height)^0.725^ × 0.0007184 [11]. Postoperative renal function was assessed by eGFR calculation. We used two different equations for eGFR calculation. The first one was the Modification of Diet in Renal Disease (MDRD) equation modified for Japanese patients [12]: eGFR = 194 × serum Cr^− 1.094^ × Age^− 0.287^ × 0.739 (if female). The second one was the equation of CKD-EPI formula for white or other [13]: eGFR = 141 × (serum Cr/0.9)^− 0.411^ × (0.993)^Age^ (if male & Cr ≤0.9 mg/dL); eGFR = 141 × (serum Cr/0.9)^− 1.209^ × (0.993)^Age^ (if male & Cr > 0.9 mg/dL); eGFR = 144 × (serum Cr/0.7)^− 0.329^ × (0.993)^Age^ (if female & Cr ≤0.7 mg/dL); eGFR = 144 × (serum Cr/0.7)^− 1.209^ × (0.993)^Age^ (if female & Cr > 0.7 mg/dL). We compared preoperative CCr with two different eGFR formulas in order to decide the optimal eGFR equation for this study.

We categorized the kidney donors into two groups. Patients whose eGFR at 1 year after donation was ≥70% of their pre-donation eGFR were assigned to the “favorable renal compensation” group, and those whose eGFR at 1 year after donation was <70% of their pre-donation eGFR were assigned to the “unfavorable renal compensation” group. Delanaye P et al. conducted a literature review about post-donation renal function and mentioned that many studies reported post-donation renal function as 65–70% of the base line [14]. Therefore, we determined the threshold between the “unfavorable renal compensation” and “favorable renal compensation” groups as 70% eGFR of the base line. The investigated variables included age (years), sex (female), height (m), weight (kg), BMI (kg/m^2^), hypertension (defined as the use of antihypertensive drugs), total kidney volume (cm^3^), PKV, BSA-adjusted PKV, PKV ratio (%), preoperative serum uric acid (mg/dL), preoperative serum Cr (mg/dL), preoperative CCr (mL/min/1.73m^2^), preoperative eGFR (mL/min/1.73m^2^), eGFR at 1-year after kidney donation (mL/min/1.73m^2^), and percent change of 1-year eGFR. BMI was calculated by the following equation: BMI (kg/m^2^) = weight (kg)/(height [m])^2^.

### Statistical analyses

Results are presented as median and range of distribution for continuous variables and number and percentage for categorical variables (Table 1). Differences between the “favorable” and “unfavorable” renal compensation groups were compared using χ^2^ statistics for categorical variables and the Wilcoxon rank sum test for non-normally distributed data or a *t*-test for normally distributed data. To predict the probability of the outcome, that is “% change of 1-year eGFR” being ≥70% of pre-donation eGFR, we conducted multivariable logistic regression analysis. Coefficients, crude odds ratio (OR), adjusted OR, and Wald χ^2^ for each covariate are shown in Tables 2 and 3. Probability (*p*) values of <0.05 were considered statistically significant. In addition, we drew receiver operating characteristic (ROC) curve of the predictive equation and the area under the curve (AUC) was calculated. Statistical analysis was performed using JMP® Pro v 13.2.0 (SAS Institute, Inc., Cary, NC, USA) and statistical figures were drawn using GraphPad Prism v 5.0 (GraphPad Software, San Diego, CA, USA).

**Table 1:** Demographic and clinical characteristics of the unfavorable and favorable renal compensation cohorts

Variables	Unfavorable renal compensation (%change of 1-year eGFR <70%) (*n* = 61)	Favorable renal compensation (%change of 1-year eGFR ≥70%) (*n* = 40)	*p* value
Age (years)	59 (33–79)	56 (31–71)	0.6644
Sex (female)	31 (51%)	25 (63%)	0.3075
Height (m)	1.62 (1.47–1.78)	1.60 (1.50–1.81)	0.5389
Weight (kg)	60.2 (41.9–93.5)	55.0 (39–76.1)	0.0026
BMI (kg/m^2^)	24.0 (15.7–31.8)	21.7 (16.9–26.6)	0.0009
Hypertension	15 (25%)	3 (8%)	0.0341
Total kidney volume (cm^3^)	343.7 (215.1–494.4)	348.2 (250.9–499.8)	0.4061
Preserved kidney volume (cm^3^)	161.5 (103.6–226.8)	169.4 (124.0–235.9)	0.3667
Body surface area-adjusted preserved kidney volume (cm^3^/m^2^)	98.8 (76.2–119.8)	107.5 (85.3–138.8)	0.0009
Preserved kidney volume ratio (%)	49.2 (44.3–53.4)	49.5 (43.5–59.1)	0.3983
Preoperative serum uric acid (mg/dL)	5.4 (3.7–7.8)	5.5 (0.7–8.6)	0.5203
Preoperative serum creatinine (mg/dL)	0.79 (0.50–1.10)	0.67 (0.41–1.02)	0.0021
Preoperative creatinine clearance (mL/min/1.73 m^2^)	112.5 (78.8–168.1)	124.9 (76.1–175.4)	0.0287
Preoperative eGFR (mL/min/1.73 m^2^)	93.2 (65.5–118.9)	97.8 (81.8–129.6)	0.0014
eGFR at 1-year after kidney donation (mL/min/1.73 m^2^)	57.9 (37.6–81.3)	75.6 (59.5–121.6)	<.0001
%change of 1-year eGFR	62.9 (49.3–69.4)	75.4 (70.5–93.8)	<.0001

Data are n (%) or median (range)

**Table 2:** Multivariable logistic regression analysis to determine independent predictors for unfavorable renal compensation at 1-year post kidney donation

	β (S.E.)	Wald Chisquare	Crude OR (95% CI)	Adjusted OR (95% CI)	*p* value
Hypertension	−0.597 (0.476)	1.574	4.022 (1.082–14.950)	3.300 (0.511–21.230)	0.2096
Preoperative eGFR (mL/min/1.73 m^2^)	−0.056 (0.032)	3.105	0.933 (0.893–0.975)	0.946 (0.889–1.006)	0.0780
BMI (kg/m^2^)	0.294 (0.109)	7.228	1.285 (1.097–1.506)	1.342 (1.083–1.663)	0.0026
Body surface area-adjusted preserved kidney volume (cm^3^/m^2^)	−0.074 (0.025)	9.109	0.923 (0.891–0.970)	0.929 (0.885–0.974)	0.0009

**Table 3:** Multivariable logistic regression analysis by using the independent covariates in Table 2

	β (S.E.)	Wald Chisquare	Crude OR (95% CI)	Adjusted OR (95% CI)	*p* value
BMI (kg/m^2^)	0.309 (0.100)	9.455	1.285 (1.097–1.506)	1.361 (1.137–1.692)	0.0004
Body surface area-adjusted preserved kidney volume (cm^3^/m^2^)	−0.088 (0.024)	13.070	0.923 (0.891–0.970)	0.916 (0.869–0.957)	<.0001

## Results

### Comparison of preoperative eGFR and CCr

**Fig. 2:**
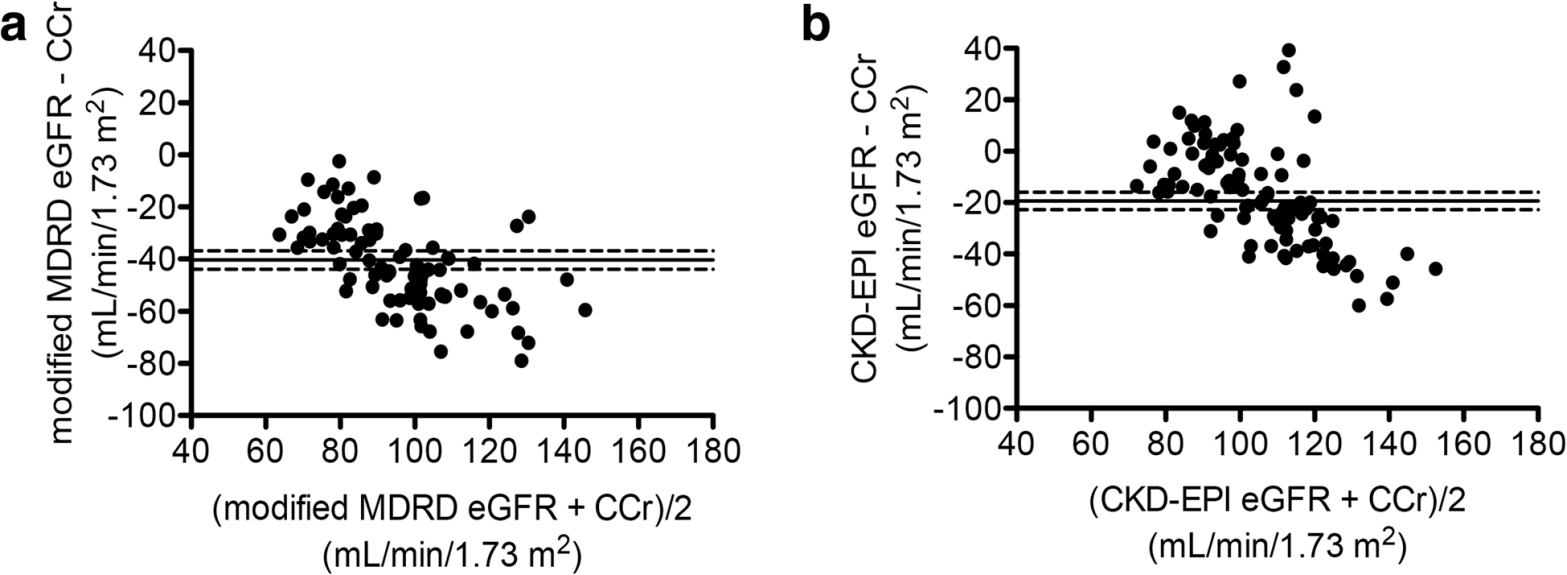
Bland–Altman plots for the comparison between preoperative CCr and two different eGFR equations. **a** Comparison between preoperative CCr and modified MDRD eGFR for Japanese. **b** Comparison between preoperative CCr and CKD-EPI eGFR. The X-axis represents the mean of each eGFR and CCr. The Y-axis represents the difference between CCr and each eGFR. Dots represent data of each donor. The horizontal continuous lines represent the average of the difference between CCr and eGFR, and dashed lines represent 95% CI

We first assessed the correlation between preoperative eGFR of two different equations (modified MDRD equation for Japanese and CKD-EPI equation) and CCr by using Bland-Altman plot analysis. The mean difference between CCr and modified MDRD eGFR was − 40.4 mL/min/1.73m^2^ (95% confidence interval [CI]: − 44.0 to − 36.9 mL/min/1.73m^2^) (Fig. 2a). The correlation coefficient of this analysis was 0.658. Meanwhile, the mean difference between CCr and CKD-EPI eGFR was − 19.4 mL/min/1.73m^2^ (95% CI: − 22.8 to − 16.0 mL/min/1.73m^2^) (Fig. 2b). The correlation coefficient of this analysis was 0.725. These results demonstrated that modified MDRD eGFR more underestimated CCr in each individual. Therefore, we decided to use the CKD-EPI equation for eGFR calculation in our cohort.

### Univariate analysis of each variable between the “favorable” and “unfavorable” renal compensation groups

Table 1 demonstrates the characteristics of 101 patients who were assigned in the “unfavorable renal compensation” or “favorable renal compensation” groups by using modified MDRD equation of eGFR for the Japanese population (40 and 61 in the “favorable” and “unfavorable” renal compensation groups, respectively). Median weight and BMI were significantly higher in the “unfavorable” than in the “favorable” renal compensation groups (60.2 vs. 55.0 kg, *p* = 0.0026 and 24.0 vs. 21.7 kg/m^2^, *p* = 0.0009, respectively). The ratio of donors with hypertension was significantly higher in the “unfavorable” than in the “favorable” renal compensation groups (25 vs. 8%, *p* = 0.0341, respectively). Preoperative characteristics, including age, sex, height, PKV ratio, and serum uric acid levels, were not significantly different between the two groups by univariate statistical analyses.

Although total kidney volume and PKV were not statistically different between the groups, BSA-adjusted PKV was significantly higher in the “favorable” renal compensation group than in the “unfavorable” one (107.5 vs. 98.8 cm^3^, *p* = 0.0009).

Median preoperative Cr was significantly higher in the “unfavorable” renal compensation group than in the “favorable” one (0.79 vs. 0.67 mg/dL, *p* = 0.0021, respectively). Median preoperative CCr and eGFR were significantly lower in the “unfavorable” renal compensation group than in the “favorable” one (112.5 vs. 124.9 mL/min/1.73 m^2^, *p* = 0.0287 and 93.2 vs. 97.8 mL/min/1.73 m^2^, *p* = 0.0014, respectively).

Median eGFR at 1 year after kidney donation and median percent change of 1-year eGFR were significantly higher in the “favorable” renal compensation group than in the “unfavorable” one (75.6 vs. 57.9 mL/min/1.73m^2^, *p* <.0001 and 75.4% vs. 62.9%, *p* <.0001, respectively).

### Independent predictors for unfavorable renal compensation at 1 year after kidney donation

We subsequently performed multivariable logistic regression analysis using significant covariates that were obtained in the univariate analysis (hypertension, preoperative eGFR, BMI, and BSA-adjusted PKV) to determine the independent predictors for unfavorable renal compensation at 1 year after kidney donation. We did not include weight, Cr, and CCr because these covariates potentially interact with BMI or preoperative eGFR.

When hypertension, preoperative eGFR, BMI, and BSA-adjusted PKV were adjusted together, it became evident that BMI (adjusted OR, 1.342; 95% CI, 1.083–1.663; *p* = 0.0026), and BSA-adjusted PKV (adjusted OR, 0.929; 95% CI, 0.885–0.974; *p* = 0.0009) were independent predictors for eGFR decline >30% at 1 year after kidney donation (Tables 2). This result indicated that hypertension and preoperative eGFR were not independent predictors for unfavorable renal compensation. Tests by entering interaction terms for each covariate into the logistic regression analysis revealed no interaction among hypertension, preoperative eGFR, BMI, and BSA-adjusted PKV (data not shown). When BMI and BSA-adjusted PKV were adjusted together, each coefficient was 0.309 and − 0.088, respectively (Table 3). Therefore, the logit probability for the outcome of unfavorable renal function compensation was as follows: logit (*p*) = (0.309 × BMI) + (− 0.088 × BSA-adjusted PKV) + 2.443.

A whole model test (comparing the model in Table 3 to the model including intercept only) denoted that the model in Table 3 was significant (χ^2^, 26.838, *p* <.0001). A lack of fit test indicated that the model contained sufficient numbers of covariates and the linear model appropriately fit the data (χ^2^, 92.231, *p* = 0.2775).

### Diagnostic accuracy evaluation of the predictive model and cut-off values of BMI and BSA-adjusted PKV

**Fig. 3:**
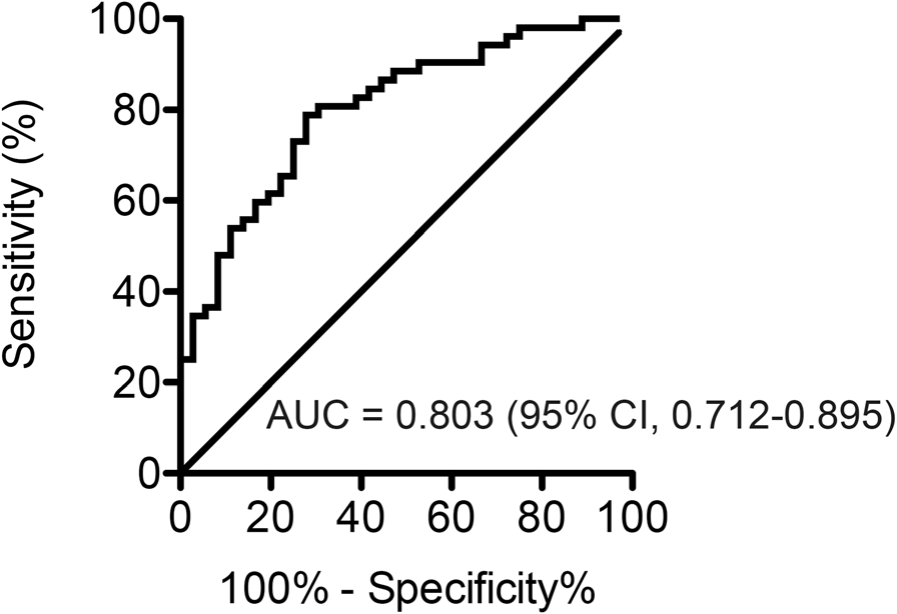
The diagnostic accuracy evaluation for the predictive equation using BMI and BSA-adjusted PKV by ROC curve. AUC and its 95% CI are also shown

The ROC analysis showed a strong diagnostic accuracy of the predictive equation using independent predictors, BMI and BSA-adjusted PKV to predict the donors with unfavorable renal functional compensation (AUC, 0.803; 95% CI, 0.712–0.895; Fig. 3). The ROC curve also identified the optimal cut-off values of 23.4 kg/m^2^ for BMI and 107.3 cm^3^/m^2^ for BSA-adjusted PKV.

## Discussion

Our findings demonstrated that BMI, and BSA-adjusted PKV calculated by the 3-D reconstructed image using a thin-sliced CT scan were significant preoperative predictors for patients who lose >30% of eGFR at 1 year after kidney donation. Moreover, multivariable logistic regression analysis revealed that pre-donation eGFR (CKD-EPI) was not an independent predictor for the magnitude of renal function compensation after kidney donation. PKV may surpass the baseline eGFR in the point to give information for predicting the magnitude of renal function compensation after kidney donation.

The significance of preserved kidney volumetry by CT scan in kidney transplant donors has been argued mostly in terms of a potential substitute for nuclear renography [10, 15–18], a predictor for CKD stage 3 after donation [19], or a predictor for post-donation eGFR [20, 21]. This study differs from the previous studies because we investigated whether BSA-adjusted PKV is useful in the prediction of unfavorable renal functional compensation, in other words unfavorable adaptive hyperfiltration, not post-donation renal function. Studies about the usefulness of CT volumetry as a predictor for the magnitude of adaptive hyperfiltration after kidney donation are very rare. Jeon et al. [22] reported that lower preoperative volume of the remaining kidney was an independent predictor of delayed renal function recovery after kidney donation. However, they defined the cohort of delayed renal function recovery as that having eGFR <60 mL/min/1.73 m^2^ without comparison with preoperative renal function, which may lack the accuracy in estimating compensatory hypertrophy. Lenihan et al. [23] reported the very interesting study about the mechanism of adaptive hyperfiltration after kidney donation in terms of glomerular dynamics. Their model explains the glomerular filtration by introducing four determinants, which are renal plasma flow (RPF), glomerular transcapillary hydraulic pressure, plasma oncotic pressure, and glomerular-filtering surface area. They showed that adaptive hyperfiltration was maintained at a constant level for many years after kidney donation, which, in other words, is benign hyperfiltration after living kidney donation [24]. They found that the increase in RPF and glomerular-filtering surface area, in other words glomerular hypertrophy or glomerular volume increase, was constantly observed in parallel for 6–8 years after kidney donation. Their findings well explained how post-donation hyperfiltration is maintained without the relation to the excessive increase in glomerular hydraulic pressure. Moreover, they demonstrated that glomerular-filtering surface area is significantly correlated with renocortical volume. Inferring the evidence above, the higher kidney volume is related to higher glomerular-filtering surface area, which in turn may lead to higher magnitude of adaptive hyperfiltration after kidney donation.

Obesity has been suggested as a risk factor for kidney function insufficiency after donor nephrectomy [7, 25, 26] or unilateral nephrectomy in non-donor patients [27, 28]. However, the underlying mechanisms of kidney function insufficiency caused by obesity in patients undergoing nephrectomy have not been clearly explained. Weisinger et al. [29] first suggested obesity-related glomerulopathy by detecting focal segmental glomerulosclerosis and reversible nephrotic range proteinuria in extremely obese patients. Whether the kidneys from obese donors, who, other than having obesity, are relatively closer to the healthy cohort than the cohort with obesity-related glomerulopathy, have some histologic damage is of great concern. Rea et al. [30] compared implant biopsy specimens from obese and nonobese donors and did not identify any difference in chronic histologic damage between these two groups. On the other hand, Ohashi et al. [26] reported more prominent chronic histologic changes in implant biopsy specimens from donors with metabolic syndrome. Interestingly, Rea et al. [30] noted that the biopsy specimens from obese donors contained larger glomerular planar surface area compared to those from the non-obese cohort and they also detected dilated tubules in obese donors. Another report also showed that BMI is correlated positively with glomerular cross-sectional area [31]. These findings may reflect the phenomenon that the kidneys in obese donors are relatively shifted to the hyperfiltration state. This may explain why adaptive hyperfiltration after kidney donation in obese donors is less satisfactory than that in non-obese donors. In fact, Choi et al. [28] demonstrated that the magnitude of adaptive hyperfiltration was lower in extremely obese non-donor patients who underwent nephrectomy.

The reason why known predictive factors, such as age or hypertension were not significant covariates in the multivariable logistic regression analysis is another concern. Several studies suggested that age and pre-donation hypertension were negatively correlated with post-donation renal function [6–8]. On the other hand, some reports suggested that relatively high-risk donors, including those with older age or preexisting hypertension, exert similar post-donation renal function or compensatory hypertrophy compared to low-risk donors [32, 33]. One speculation is that follow-up of up to 5 years after kidney donation may be relatively short when evaluating the effect of these risk factors on post-donation renal compensation in the cohort of strictly-selected living kidney donors [32, 33]. They are relatively younger and less hypertensive compared to patients undergoing nephrectomy for cancers or reasons other than kidney donation. Although preserved kidneys in such high-risk donors exert favorable compensatory hypertrophy or adaptive hyperfiltration capacity after kidney donation, the more prominent glomerulopenia was actually observed [34, 35]. The investigators considered that such high-risk donors need careful long-term follow-up. Fesler et al. [36] argued from a different viewpoint. They noted that increased arterial stiffness measured by carotid-to-femoral pulse wave velocity is an independent risk factor for decreased post-donation compensatory hyperfiltration, although baseline BP was not correlated with adaptive glomerular hyperfiltration. Further validation studies are required to evaluate if arterial stiffness reflects a decreased capacity in baseline renal hemodynamics.

We must acknowledge some limitations of our study. Our study had a single-institution retrospective design and sample size was relatively small. The 1-year follow-up was relatively short. However, the aim of this study is to select eligible candidates among marginal living donors by evaluating a potential of renal function compensation in a short term after the operation. Living kidney donation from donor candidates with marginal renal function and unfavorable compensation in a short term after kidney donation should be prohibited. In our study, donors with hypertension were defined as those taking antihypertensive medication and their BP was already corrected in the normal range. This might weaken the power of hypertension as a covariate in our study. However, it should be considered that the studies using kidney transplant donors tend not to include untreated or drug-resistant hypertensive patients. In addition, evaluation of renal function should be done basically by using measured GFR, although we had to use eGFR for assessment of renal function.

## Conclusions

Before kidney donation, we can predict which candidates have a potential of unfavorable renal function compensation using preoperative BMI and calculation of PKV. Predicting the magnitude of renal function compensation before operation may be helpful to assess the eligibility of kidney donor candidates when their baseline kidney function is marginal.

## Abbreviations

3-D: 3-dimensional
AUC: Area under the curve
BMI: Body mass index
BP: Blood pressure
BSA: Body surface area
CCr: Creatinine clearance
CI: Confidence interval
CKD: Chronic kidney disease
Cr: Creatinine
CT: Computed tomography
eGFR: Estimated glomerular filtration rate
ESRD: End-stage renal disease
MDRD: Modification of Diet in Renal Disease
OR: Odds ratio
PKV: Preserved kidney volume
ROC: Receiver operating characteristic
RPF: Renal plasma flow
